# A71 SIGNIFICANT RACIAL/ETHNIC DIFFERENCES EXIST IN THE RECEIPT OF IBD-RELATED SURGERY: A SYSTEMATIC REVIEW AND META-ANALYSIS

**DOI:** 10.1093/jcag/gwac036.071

**Published:** 2023-03-07

**Authors:** T Chhibba, P Tandon, N Natt, G Brar, G Malhi, G Nguyen

## Abstract

**Background:**

Patients with inflammatory bowel disease (IBD) may require surgical intervention for management of their disease. There is a rising incidence of IBD in racial and ethnic minorities but studies regarding healthcare utilization patterns in these populations have yielded variable results.

**Purpose:**

We aimed to examine the differences in surgical rates of ethnic and racial groups compared to White patients with IBD.

**Method:**

Electronic databases were searched through December 20, 2021. Studies that compared ulcerative colitis (UC) or Crohn’s disease (CD) surgery rates between different racial/ethnic groups were included. Both pediatric and adult studies were included. Pooled event rates were generated and p-value < 0.05 was considered statistically significant in generating odds ratios (OR) with 95% confidence interval (CI). We also compared differences in disease location, phenotype, and IBD-medication exposure amongst different groups included.

**Result(s):**

Forty-one studies stratified rates of IBD-related surgeries by race or ethnicity (n=1,094,693 patients). Black patients were less likely to undergo IBD-related surgeries compared to White patients (pooled OR 0.70, 95% CI, 0.55-0.89, I2=87.0%). Black patients were also less likely compared to White patients to undergo an emergent colectomy with an incidence rate ratio of 0.43 (95% CI, 0.32-0.58). Furthermore, Hispanic patients were less likely to undergo a CD-related surgery (pooled OR 0.57, 95% CI, 0.48-0.68, I2=0%) compared to White patients. Finally, Asian patients had no significant difference in likelihood of CD-related and UC-related surgeries compared to White patients. Black patients were more likely to have perianal disease (pooled OR 1.40, 95% CI, 1.06-1.86), I2=58.2%) but otherwise disease characteristics and phenotypes were similar across all populations compared to Caucasians.

**Conclusion(s):**

Black and Hispanic patients with IBD are less likely to have surgery, including emergent surgery, for IBD compared to White patients with IBD, despite similar disease phenotype characteristics. Disparities in access to care may be contributory toward these findings and efforts should be made to provide equitable care to all persons living with IBD, regardless of race and ethnicity.

**Please acknowledge all funding agencies by checking the applicable boxes below:**

Other

**Please indicate your source of funding below:**

Nil

**Disclosure of Interest:**

None Declared

